# Differences in consumer use of food labels by weight loss strategies and demographic characteristics

**DOI:** 10.1186/s12889-015-2651-z

**Published:** 2015-12-22

**Authors:** Sara N. Bleich, Julia A. Wolfson

**Affiliations:** Department of Health Policy and Management, Johns Hopkins Bloomberg School of Public Health, Baltimore, USA

**Keywords:** Fast food labels, Packaged food labels, Weight loss behaviors

## Abstract

**Background:**

Little is known about national patterns in the use of fast food and packaged food labels among adults by weight loss strategies and demographic characteristics.

**Methods:**

We analyzed the Consumer Behavior Module in the National Health and Nutrition Examination Survey 2007–2010 among adults (*N* = 9,690). For each of the outcome variables – use of packed food and fast food menu labels – multiple logistic regressions were used to adjust for potential differences in population characteristics by weight loss activities and demographic characteristics.

**Results:**

Overall, 69 percent of adults reported they would use fast food information and 76 percent reported using the nutrition facts panel on packaged foods. Adults trying to lose weight had a greater likelihood of reporting use of nutrition information to choose fast foods (OR = 1.72; 95 % CI: 1.29, 2.29) and using the nutrition facts panel on food labels (OR = 1.92; 95 % CI: 1.60, 2.30). Black and Hispanic adults were more likely to report using ingredients lists on packaged foods compared to Whites (White −63 %, Black/Hispanic −68 %, *p* < 0.05).

**Conclusion:**

Regardless of weight loss activities or demographic characteristics, a majority of adults report they would use fast food nutrition information.

**Electronic supplementary material:**

The online version of this article (doi:10.1186/s12889-015-2651-z) contains supplementary material, which is available to authorized users.

## Background

The federal government has mandated nutritional labeling on the majority of packaged foods since 1990 through the Nutritional Labeling and Education Act of 1990 [1]. More recently, the 2010 *Patient Protection and Affordable Care Act* (ACA) required restaurants and similar food service establishment chains with 20 or more locations in the United States to provide calorie information on their menus and menu boards with a statement addressing daily recommended caloric intake [2]. The Food and Drug Administration has finalized the menu labeling regulations for chain restaurants which are expected to be implemented in December 2016. Both labeling schemes have the goal of helping consumers make healthier or lower calorie dietary choices and potentially lessen the burden of the obesity epidemic which currently affects one-third of American adults [3] and costs $147 billion in U.S. health spending annually [4].

Generally, evidence suggests that consumers perceive nutrition labels to be a credible source of information and many consumers report using nutrition labels to guide their food selections [5]. In addition, the use of nutrition labels is associated with a healthier diet [5]. For instance, one study found that when consumers are presented with calorie information they chose the high-calorie items approximately a third less often [6], and another study found that when exposed to calorie information at the point-of-purchase, people buy food with fewer calories [7]. Calorie information in fast food restaurants or on packaged foods, therefore, has the potential to have a considerable impact on consumer behavior – a particularly important area of research given consumers’ significant underestimation of the amount of calories in the foods they consume [6, 8].

However, consumer use of nutrition labels is not uniform. Females, higher income individuals, health conscious individuals, and more educated individuals are more likely to report looking at food packaging nutrition labels [9] – groups which are generally at lower risk for obesity [10]. Prior nutrition knowledge and a desire to eat healthy are also associated with using food packaging nutrition labels [11]. In addition, adults trying to lose weight may be more likely to use food labels as a guide to healthier choices. Prior research suggests that among adults trying to lose weight, two of the most common weight-loss strategies are eating fewer calories and eating less fat [12].

The recent incorporation of the Consumer Behavior Module into the National Health and Nutrition Examination Survey (NHANES), which asks about the use of both packaged and fast food menu labels, presents a unique opportunity for a more comprehensive understanding of national use of nutrition labels in both fast food restaurants and on packaged foods among a national sample of U.S. adults. The primary aim of this study was to describe patterns in the use of fast food menu labeling and packaged food labels among adults by weight loss strategies. The secondary aim was to examine whether use of fast food menu labeling and packaged food labels differed by demographic characteristics, particularly those associated with obesity risk (socioeconomic status and race/ethnicity). We hypothesized that food label use would be higher among adults trying to lose weight compared to those who were not. We further hypothesized that food label use would be higher among groups at lower risk for obesity.

## Methods

### Data and design

Data was obtained from the nationally representative NHANES (2007–2010). The NHANES is a population-based survey designed to collect information on the health and nutrition of the U.S. population. Participants were selected based on a multi-stage, clustered, probability sampling strategy. Our analysis combined the continuous NHANES data collection (2007–2010) to look at overall patterns during that time period. A complete description of data-collection procedures and analytic guidelines are available elsewhere (www.cdc.gov/nchs/nhanes.htm).

### Study sample

The study sample consisted of adults ages 20 and older who completed the dietary interview in the NHANES. Survey respondents were excluded if they were pregnant or had diabetes at the time of data collection or if their dietary recall was incomplete or unreliable (as determined by the NHANES staff). The final analytic sample included 9,690 adults. Consent for the study was obtained by the NHANES staff.

### Measures

#### Packaged food labels

The Consumer Behavior Module in the NHANES asks detailed questions about use of packaged food labels. Each respondent was asked the following: 1) How often do you use the nutrition facts panel?; 2) How often do you use the list of ingredients?; 3) How often do you use the size of serving?; 4) How often do you use the health claim?; and 5) How often do you use percent daily value? Response categories ranged from: always, most of the time, sometimes, rarely, never, and never seen. We characterized respondents as ‘using’ each packaged food label component if they responded always, most of the time or sometimes based on the cut-points in the data. Of note, only those respondents who completed a dietary interview portion of the NHANES were eligible for the Consumer Behavior Module.

#### Fast food menu labels

The Consumer Behavior Module in the NHANES also asks detailed questions about use of fast food menu labels. Each respondent was asked the following: 1) Whether they saw nutrition information on fast food menus (response categories – yes/no); 2) Whether they used nutrition information to choose fast foods (response categories – yes/no); and 3) Whether they would use fast food nutrition information (response categories – often, sometimes, rarely, never). We characterized respondents as ‘would use’ fast food nutritional information if they responded often or sometimes based on the cut-points in the data.

#### Weight loss activities

Respondents were classified as pursuing weight loss activities if they answered ‘yes’ to the survey question “During the past 12 months, have you tried to lose weight?” If they answered affirmatively, they were asked a series of detailed questions about the types of weight loss activities they were engaged in which were categorized into four groups: dietary changes (e.g., ate less, switched to lower calorie foods), physical activity (e.g., exercised), commercial diets (e.g., weight loss program) and other (e.g., prescription diet pills, use of laxatives). Virtually all respondents who reported that they were trying to lose weight engaged in at least one weight loss activity (99 %) Of note, prescription diet pills and non-prescription supplements were not placed in a standalone group due to low levels of reported utilization (N, prescription = 96; N, non-prescription = 230).

#### Demographic characteristics

Race was characterized into three mutually exclusive categories: 1) non-Hispanic White, non-Hispanic Black and Mexican American. Education was categorized into three mutually exclusive categories: 1) less than high school; 2) high school (or GED) and 3) more than high school.

### Analysis

All analyses were weighted to be representative of the general population and conducted using STATA, version 12 (StataCorp, L.P., College Station, TX) to account for the complex sampling structure. For each of the outcome variables – use of packed food and fast food menu labels – multiple logistic regressions were used to adjust for potential differences in population characteristics by weight loss activities and demographic characteristics, including gender and household income. Using post-estimation commands, we calculated the predicted probability of using fast food menu labels and packaged food labels. For all models, statistical significance was determined at *p* < 0.05. All covariates were included in the models based on the literature regardless of statistical significance [9, 11, 12].

## Results

The characteristics of the NHANES 2007–2010 sample are presented in Table 1, overall and by weight loss effort. The categories of weight loss effort had comparable distributions of race/ethnicity. The category of adults pursing weight loss activities had more women, young adults (20–44), more educated (more than high school education), married, overweight and obese and higher income adults (*p* < 0.05).

**Table 1:** Characteristics of US adults (aged ≥20 y) in the National Health and Nutrition Examination Survey (NHANES) 2007-2010^a^, overall and by weight loss activities

		Weight loss efforts		
	Total	Pursuing weight loss activities	No weight loss activities	*P for diff*
	N	N(%)	N(%)	
Total	9690	3842 (43)	5848 (57)	
Sex				
Male	4772	1536 (40)	3236 (54)	<0.001
Female	4918	2306 (60)	2612 (46)	
Race-ethnicity				
Non-Hispanic white	4772	1891 (75)	2883 (74)	0.50
Non-Hispanic black	1764	696 (11)	1068 (11)	
Mexican American	2706	1092 (13)	1614 (14)	
Age				
20–44 y	4382	1899 (52)	2483 (48)	< 0.001
45–64 y	3180	1327 (37)	1853 (35)	
≥ 65 y	2128	616 (11)	1512 (18)	
Education				
Less than high school	2689	811 (14)	1878 (22)	< 0.001
High school (or GED)	2328	872 (22)	1456 (25)	
More than high school	4661	2158 (64)	2503 (53)	
Marital status				
Currently married	5034	2044 (57)	2990 (53)	0.03
Previously married	2116	791 (16)	1325 (19)	
Living with a partner	777	277 (7)	500 (9)	
Never married	1760	729 (20)	1031 (20)	
Bodyweight^b^				
Healthy	2762	561 (18)	2201 (42)	< 0.001
Overweight	3374	1381 (37)	1993 (34)	
Obese	3304	1871 (45)	1433 (24)	
Poverty income ratio				
< 130 % FPL	2778	955 (18)	1923 (24)	< 0.01
≥ 130 % FPL	6059	2606 (82)	3453 (76)	

Note: *P*-value for difference is based on chi-squared test

^a^ Percentage of US population estimated with survey weights to adjust for unequal probability of sampling

^b^ Healthy weight [BMI (kg/m^2^) 18.5–24.99], Overweight (BMI 25–29.99), Obese (BMI ≥ 30)

### Overall label use

Table 2 reports the percentage of adults using fast food and packaged food labels. Overall, 22 % of adults reported seeing nutritional information at fast food restaurants, nine percent said they used the information to choose fast foods, and 69 % said they would use fast food information. Roughly half to three-fourths of adults reported using labels on packaged foods: nutritional facts panel (76 %), ingredient list (64 %), serving size (62 %), percent daily value (56 %), and health claims (66 %). Adults pursuing weight loss activities were significantly more likely to use fast food and packaged food labels for each outcome with the exception of seeing the information on fast food menus: use nutrition information to choose fast food (11 % vs. 7 %, *p* = 0.001), would use fast food nutritional information (73 % vs. 65 %, *p* = 0.001), use nutritional facts panel on food label (82 % vs. 72 %, *p* < 0.001), use ingredient list on food label (68 % vs. 61 %, *p* < 0.001), use serving size on food label (67 % vs. 58 %, *p* < 0.001), use percent daily value (60 % vs. 53 %, *p* < 0.001), and use health claims on food packages (69 % vs. 62 %, *p* < 0.001).

**Table 2:** Percentage of U.S. adults (ages 20+) using fast food menu labels and packaged food labels, overall and by weight loss activities, NHANES 2007–2010^a^

		Weight loss activities	
	ALL	Pursuing weight loss activities	No weight loss activities
	Mean ± SEM	Mean ± SEM	Mean ± SEM
Fast Food Menu Label^b^			
Saw nutrition info on fast food menu (*N* = 1,332)	22 ± 1	22 ± 1	22 ± 2
Used nutrition info to choose fast foods (*N* = 599)	9 ± 1	11 ± 1*	7 ± 1
Would use fast food nutrition info (*N* = 4,564)	69 ± 1	73 ± 1*	65 ± 1
Food Labels			
Use nutrition facts panel on food label (*N* = 5,926)	76 ± 1	82 ± 1*	72 ± 1
Use of ingredient list on food label (*N* = 5,241)	64 ± 1	68 ± 1*	61 ± 1
Use of serving size on food label (*N* = 4,967)	62 ± 1	67 ± 1*	58 ± 2
Use of percent daily value on food label (*N* = 4,653)	56 ± 1	60 ± 2*	53 ± 1
Use of health claims on food packages (*N* = 5,388)	66 ± 1	69 ± 1*	62 ± 1

Note: Multivariate regression was used to adjust for sex, race/ethnicity, age, education, marital status, poverty and body-weight category; S.E.M. = standard error of the mean

*Different from those not pursuing weight loss activities at *p* < 0.05

^a^ standard errors <0.5 were rounded to 0

^b^ Only respondents who complete a dietary interview in the mobile examination center (MEC) are eligible for the Flexible Consumer Behavior Survey (FCBS) module resulting in high baseline missingness for these variables. Appropriate survey weights were used which adjust for the additional non-response for these variables

### Label use by weight loss activities

Table 3 presents the adjusted associations between use of food labels (fast food menus and packaged foods) and weight loss activities. Compared to adults who did not engage in any weight loss activities, those who did attempt to lose weight had greater odds of reporting use of nutrition information to choose fast foods (OR = 1.72; 95 % CI: 1.29, 2.29) as well as using fast food nutrition information (OR = 1.49; 95 % CI: 1.20, 1.84), nutrition facts panel on food labels (OR = 1.92; 95 % CI: 1.60, 2.30), ingredient list on food labels (OR = 1.39; 95 % CI: 1.20, 1.61), serving size on food labels (OR = 1.50; 95 % CI: 1.25, 1.80), percent daily value on food labels (OR = 1.35; 95 % CI: 1.17, 1.57), and health claims on food packages (OR = 1.39; 95 % CI: 1.18, 1.63), after adjustment for covariates.

**Table 3:** Adjusted association between use of food labels and weight loss activities, NHANES 2007–2010

	Weight Loss Activities				
	Any	Dietary changes	Physical activity	Commercial diets	Other
	*N* = 3,842	*N* = 3,480	*N* = 2,310	*N* = 665	*N* = 402
	OR [95 % CI]	OR [95 % CI]	OR [95 % CI]	OR [95 % CI]	OR [95 % CI]
Fast Food Menu Label					
Saw nutrition info on fast food menu	1.00 [0.77, 1.29]	0.96 [0.70, 1.32]	1.02 [0.77, 1.34]	1.43* [1.01, 2,02]	1.14 [0.75, 1.73]
Used nutrition info to choose fast foods	1.72* [1.29, 2.29]	1.43* [1.06, 1.93]	1.14 [0.81, 1.59]	1.94* [1.31, 2.87]	1.09 [0.66, 1.79]
Would use fast food nutrition info	1.49* [1.20, 1.84]	1.15 [0.97, 1.38]	1.40* [1.09, 1.79]	1.50* [1.15, 1.96]	1.21 [0.86, 1.71]
Food Labels					
Use nutrition facts panel on food label	1.92* [1.60, 2.30]	1.40* [1.11, 1.78]	1.64* [1.22, 2.21]	1.53* [1.11, 2.11]	0.91 [0.64, 1.31]
Use of ingredient list on food label	1.39* [1.20, 1.61]	1.33* [1.06, 1.66]	1.20 [0.99, 1.44]	0.77* [0.60, 0.98]	0.81 [0.63, 1.04]
Use of serving size on food label	1.50* [1.25, 1.80]	1.11 [0.92, 1.34]	1.34* [1.05, 1.71]	1.56* [1.16, 2.09]	1.11 [0.84, 1.46]
Use of percent daily value on food label	1.35* [1.17, 1.57]	1.02 [0.86, 1.20]	1.56* [1.30, 1.88]	0.85 [0.66, 1.09]	1.06 [0.78, 1.43]
Use of health claims on food packages	1.39* [1.18, 1.63]	1.28* [1.02, 1.60]	1.08 [0.88, 1.34]	0.96 [0.71, 1.28]	1.18 [0.85, 1.64]

Note: Multivariate regression was used to adjust for sex, race/ethnicity, age, education, marital status, poverty, body-weight category and engagement in weight loss activities. The categories of weight loss activities included the following specific activties: dietary changes (e.g., ate less, switched to lower calorie foods), physical activity (e.g., exercised), commercial diets (e.g., weight loss program) and other (e.g., prescription diet pills, use of laxatives)

*Odds Ratio significant at *p* < 0.05

The other columns present the likelihood of food label use associated with each type of weight loss activity after adjusting for all other weight loss activities. Adults who made dietary changes to lose weight had greater adjusted odds of using nutrition information to choose fast foods (OR = 1.43; 95 % CI: 1.06, 1.93), using the nutrition facts panel on food labels (OR = 1.40; 95 % CI: 1.11, 1.78), using the ingredient list on food labels (OR = 1.33; 95 % CI: 1.06, 1.66), and using health claims on food packages (OR = 1.28; 95 % CI: 1.02, 1.60). Adults who used physical activity to lose weight had greater adjusted odds of reporting they would use nutrition information at fast food restaurants (OR = 1.40; 95 % CI: 1.09, 1.79), using the nutrition facts panel on food labels (OR = 1.64; 95 % CI: 1.22, 2.21), using the serving size on food labels (OR = 1.34; 95 % CI: 1.05, 1.71), and using the percent daily value on food labels (OR = 1.56; 95 % CI: 1.30, 1.88). Adults who used commercial diets to lose weight had greater adjusted odds of reporting they saw nutrition information on fast food menus (OR = 1.43; 95 % CI: 1.01, 2,02), using nutrition information to choose fast foods (OR = 1.94; 95 % CI: 1.31, 2.87), reporting they would use nutrition information at fast food restaurants (OR = 1.50; 95 % CI: 1.15, 1.96), using the nutrition facts panel on food labels (OR = 1.53; 95 % CI: 1.11, 2.11) and using the serving size on food labels (OR = 1.56; 95 % CI: 1.16, 2.09). Adults who used commercial diets to lose weight had lower odds of using the ingredient list on food labels (OR = 0.77; 95 % CI: 0.60, 0.98). We observed no significant differences in the use of food labels among adults engaging on other types of weight loss activities.

### Fast food menu label use by demographic characteristics

**Fig. 1:**
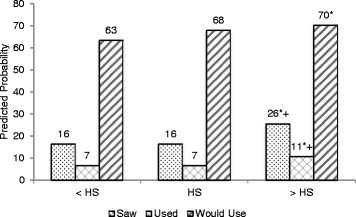
Predicted probability of seeing, using or reporting one would use fast food menu labels by education level. NHANES 2007–2010^1^

Figure 1 presents the predicted probability of using fast food menu labels by education. Higher education attainment (more than a high school diploma) was associated with a significantly higher probability of seeing, using, and reporting that one would use fast food menu labels: saw fast food menu labels (high school or less: 16 % vs. more than high school: 26 %, *p* = 0.001); using fast food menu labels (high school or less: 7 % vs. more than high school: 11 %, *p* = 0.03); would use fast food menu labels (less than high school: 63 %, more than high school: 70 %, *p* = 0.002).

We additionally examined differences in the predicted probability of using fast food menu labels by gender, race/ethnicity, and body weight category (not shown but available upon request). Women were more likely than men to report using fast food menu labels (11 % vs. 7 %, *p* = 0.004) and that they would use fast food menu labels (76 % vs. 68 %, *p* < 0.001). Hispanic adults were less likely than White adults to report seeing fast food menu labels (23 % vs. 17 %, *p* < 0.001). We observed no significant differences in seeing, using and reporting that one would use fast food menu labels by body weight category (healthy weight, overweight, obese).

### Packaged food label use by demographic characteristics

**Fig. 2:**
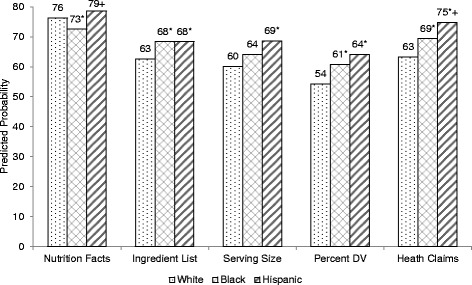
Predicted probability of using food labels by race/ethnicity. NHANES 2007-2010^1^

Figure 2 presents the predicted probability of using various types of labels on packaged foods by race/ethnicity. Compared to White adults, Black adults were more likely to use ingredient lists (68 % vs. 63 %, *p* = 0.02), percent daily value (61 % vs. 54 %, *p* = 0.004), and health claims (69 % vs. 63 %, *p* = 0.01) and less likely to use nutrition facts (73 % vs. 76 %, *p* = 0.04). Compared to White adults, Hispanic adults were more likely to use ingredient lists (68 % vs. 63 %, *p* = 0.01), serving size (69 % vs. 60 %, *p* = 0.001), percent daily value (64 % vs. 54 %, *p* = 0.001), and health claims (75 % vs. 63 %, *p* < 0.001). Compared to Black adults, Hispanic adults were more likely to use nutrition facts (79 % vs. 73 %, *p* = 0.004) and health claims (75 % vs. 69 %, *p* = 0.046).

We additionally examined differences in the predicted probability of using various types of labels on packaged foods by gender and body weight category (not shown but available upon request). Women were significantly more likely than men to use nutrition facts (82 % vs. 70 %, *p* < 0.001), ingredient lists (67 % vs. 61 %, *p* < 0.001), serving size (69 % vs. 54 %, *p* < 0.001), percent daily value (58 % vs. 54 %, *p* = 0.003), and heath claims (70 % vs. 60 %, *p* < 0.001).

The results related to education suggest that individuals with more than a high school education were significantly more likely than those with less than a high school education to use nutrition facts (79 % vs. 73 %, *p* = 0.002), and to use ingredient lists (66 % vs. 61 %, *p* = 0.04). These same individuals were also more likely than those with a high school education to use nutrition facts (79 % vs. 72 %, *p* < 0.001), ingredient lists (66 % vs. 60 %, *p* = 0.004), and serving size information (63 % vs. 59 %, *p* = 0.03).

We observed no significant differences in use of packaged food labels by body weight category. Additional file 1: Tables S1 and S2 reports the full regression results for all food label use outcomes.

## Discussion

The goal of this study was to describe patterns in the use of fast food menu labeling and packaged food labels among adults by weight loss strategies and demographic characteristics. We found that consumer use of fast food menu labels and packaged food labels is high with roughly a half to three-fourths of American adults reporting that they use this information. Consistent with our first study hypothesis, we found that label use is higher among adults engaging in weight loss activities. Results for our second study hypothesis were mixed; food label use was higher among some groups at lower risk for obesity (e.g., those with higher educational attainment) but not among other groups at lower risk for obesity (e.g., Whites).

These results provide modest support to the evidence base suggesting that menu labeling may positively impact consumer behavior by encouraging lower calorie [13, 14] or healthier food purchases [15]. Although the literature related to menu labeling and consumer purchases is mixed with several systematic reviews of the impact of menu labeling on calorie purchases finding inconsistent or null results [16–19]. Our finding of *higher* label use among Black and Hispanic adults is notable given prior research suggesting lower label use among groups with lower socioeconomic status [5]. However, it is consistent with our prior research suggesting that the federal calorie posting requirement in chain restaurants may be relatively more salient among Black and Hispanic sub-groups [20]. One key reason for differing results related to race/ethnicity may be due to methodological differences in data collection; some studies observe actual purchasing behavior in response to labels whereas others ask respondents to report how they might react in response to labels.

Interestingly, we found that fast food and packaged food label use is highest among those adults using commercial diets to lose weight. This suggests that the education individuals receive in commercial programs may be more effective at encouraging attention towards food labels than self-directed efforts. These findings may be particularly relevant to the SNAP educational program (SNAP-Ed) which recently expanded its nutrition education efforts to focus on the problem of obesity. Findings from this study may also be relevant to the recent changes to Medicaid under the *Affordable Care Act (ACA)*, particularly because the low-income Americans covered by this program are more likely to be obese [21]. The ACA gives state Medicaid programs the option to substantially expand coverage to low-income, nonelderly adults [22], and provides new incentives to states to cover obesity-related services [23].

More research is needed to better understand patterns of fast food menu labels and packaged food labels, particularly among groups at highest risk for obesity. Given our finding that most adults report they would use fast food nutrition information, regardless of whether they are trying lose weight, more research is needed to identify the most effective way of communicating this information. Past research suggests that use of absolute calorie information for restaurant items (e.g., a hamburger contains 250 cal) has relatively little influence on food selection and calorie consumption [24, 16]. This may be due to difficulties in understanding the information contained in the presentation of absolute calories [25]. Studies have found that presenting calorie information as a physical activity equivalent (e.g., minutes of running required to burn off a particular food) is more effective than absolute calories [26–28]. This area of inquiry could inform the implementation of the federal mandatory menu labeling regulation. Future research should also focus on how effective education techniques from commercial weight loss programs can be incorporated into SNAP-Ed.

### Limitations

The present findings should be interpreted in light of several limitations. First, this cross sectional analysis only allows us to address associations. Second, respondents were asked to think abstractly about how they might use fast food menu labels. Given that individuals are inclined to have an optimism bias – tendency to be overly positive about the outcome of their planned actions – our results may be somewhat inflated. Third, these data were collected prior to the implementation of the ACA, so the results may differ from public perceptions once the regulations are implemented. Fourth, these data only included those adults who completed the dietary interview in the NHANES and there may be systematic differences between adults who selected to complete the dietary interview and those who did not. Fifth, because we are unable to measure community-level confounders (e.g., proximity to fast food restaurants), there may be some omitted variable bias; however we expect this would bias the results towards the null and lead to more conservative results.

## Conclusions

To conclude, American adults who are trying to lose weight are more likely to use fast food menu labels and packaged food labels. However, a majority of adults report that they would use fast food nutrition information regardless of whether they are pursing weight loss activities which suggests that the federal menu labeling requirements for chain restaurants may be useful for promoting energy balance. More research is needed to identify the most effective mode of communicating nutrition information via fast food menu labels and packaged food labels to adults, particularly those at highest risk for obesity.

## Additional file

Additional file 1:
**Table S1.** Full model results for fast food menu label use. **Table S2.** Full model results for food label use. (DOCX 25 kb)
