# Supplementary material for: Differences in consumer use of food labels by weight loss strategies and demographic characteristics
Source: BMC Public Health. 2015 Dec 22;15:1275. doi: 10.1186/s12889-015-2651-z (PMC4687126; doi:10.1186/s12889-015-2651-z)
Supplement: Additional file 1: — Table S1. Full model results for fast food menu label use. Table S2. Full model results for food label use. (DOCX 25 kb) [file 12889_2015_2651_MOESM1_ESM.docx]

**Additional file 1: Table S1. Full model results for fast food menu label use**

|  | Fast Food Menu Label Use | | |
| --- | --- | --- | --- |
|  | Saw nutrition info on fast food menu | Used nutrition info to choose fast foods | Would use fast food nutrition info |
|  | OR  [95% CI] | OR  [95% CI] | OR  [95% CI] |
| Weight loss activities |  |  |  |
| No weight loss activities [ref] | - | - | - |
| Pursued any weight loss activity | 1.00  [0.77, 1.29] | 1.72*  [1.29, 2.29] | 1.49*  [1.20, 1.84] |
| Sex |  |  |  |
| Female [ref] | - | - | - |
| Male | 0.93  [0.77, 1.12] | 0.65*  [0.49, 0.86] | 0.46*  [0.40, 0.53] |
| Race-ethnicity |  |  |  |
| Non-Hispanic white [ref] | - | - | - |
| Non-Hispanic black | 0.96  [0.79, 1.16] | 1.30  [0.96, 1.75] | 1.18  [0.95, 1.45] |
| Hispanic | 0.69*  [0.58, 0.81] | 1.10  [0.91, 1.33] | 1.08  [0.87, 1.32] |
| Age |  |  |  |
| 20-44 y [ref] | - | - | - |
| 45-64 y | 0.86  [0.69, 1.08] | 1.10  [0.86, 1.41] | 1.16  [0.99, 1.36] |
| ≥65 y | 0.65*  [0.52, 0.82] | 0.80  [0.60, 1.07] | 0.79  [0.62, 1.02] |
| Education |  |  |  |
| Less than high school [ref] | - | - | - |
| High school (or GED) | 1.00  [0.68, 1.47] | 1.00  [0.61, 1.67] | 1.23  [0.98, 1.55] |
| More than high school | 1.77*  [1.27, 2.45] | 1.70*  [1.07, 2.69] | 1.38*  [1.14, 1.67] |
| Marital status |  |  |  |
| Currently married [ref] | - | - | - |
| Previously married | 1.13  [0.90, 1.42] | 1.07  [0.79, 1.45] | 0.83  [0.67, 1.03] |
| Living with a partner | 1.03  [0.66, 1.60] | 1.05  [0.55, 2.00] | 0.98  [0.75, 1.30] |
| Never married | 1.04  [0.79, 1.36] | 1.09  [0.71, 1.67] | 0.91  [0.75, 1.09] |
| Bodyweight^2^ |  |  |  |
| Healthy [ref] | - | - | - |
| Overweight | 1.14  [0.91, 1.44] | 1.00  [0.73, 1.63] | 0.98  [0.79, 1.22] |
| Obese | 1.21  [0.94, 1.57] | 0.71*  [0.50, 0.99] | 0.95  [0.79, 1.14] |
| Poverty income ratio |  |  |  |
| < 130% FPL | 0.96  [0.75, 1.22] | 0.94  [0.71, 1.24] | 0.79*  [0.63, 0.99] |
| ≥ 130% FPL [ref] | - | - | - |

Note: Multivariate regression was used to adjust for sex, race/ethnicity, age, education, marital status, poverty, body-weight category and engagement in weight loss activities.

*Odds Ratio significant at p<0.05.

**Additional file 1: Table S2. Full model results for food label use**

|  | Food Label Use | | | | |
| --- | --- | --- | --- | --- | --- |
|  | Use nutrition facts panel | Use of ingredient list | Use of serving size | Use of percent daily value | Use of health claims |
|  | OR  [95% CI] | OR  95% CI] | OR  [95% CI] | OR  [95% CI] | OR  [95% CI] |
| Weight loss activities |  |  |  |  |  |
| No weight loss activities [ref] | - | - | - | - | - |
| Pursued any weight loss activity | 1.92*  [1.60, 2.30] | 1.39*  [1.20, 1.61] | 1.50*  [1.25, 1.80] | 1.35*  [1.17, 1.57] | 1.39*  [1.18, 1.63] |
| Sex |  |  |  |  |  |
| Female [ref] | - | - | - | - | - |
| Male | 0.49*  [0.41, 0.57] | 0.74*  [0.66, 0.84] | 0.51*  [0.44, 0.60] | 0.86*  [0.78, 0.95] | 0.62*  [0.55, 0.71] |
| Race-ethnicity |  |  |  |  |  |
| Non-Hispanic white [ref] | - | - | - | - | - |
| Non-Hispanic black | 0.81*  [0.66, 0.98] | 1.31*  [1.06, 1.63] | 1.20  [0.96, 1.50] | 1.32*  [0.10, 1.58] | 1.34*  [1.08, 1.66] |
| Hispanic | 1.15  [0.92, 1.44] | 1.31*  [1.08, 1.57] | 1.49*  [1.18, 1.88] | 1.52*  [1.21, 1.91] | 1.77*  [1.37, 2.28] |
| Age |  |  |  |  |  |
| 20-44 y [ref] | - | - | - | - | - |
| 45-64 y | 1.57*  [1.26, 1.96] | 1.97*  [1.68, 2.31] | 1.50*  [1.22, 1.85] | 1.59*  [1.32, 1.91] | 1.69*  [1.35, 2.21] |
| ≥65 y | 2.14*  [1.73, 2.64] | 3.86*  [3.13, 4.76] | 1.92*  [1.62, 2.28] | 2.24*  [1.82, 2.76] | 2.80*  [2.28, 3.44] |
| Education |  |  |  |  |  |
| Less than high school [ref] | - | - | - | - | - |
| High school (or GED) | 0.96  [0.78, 1.19] | 0.94  [0.75, 1.18] | 0.98  [0.81, 1.19] | 0.90  [0.77, 1.06] | 1.04  [0.84, 1.29] |
| More than high school | 1.49*  [1.17, 1.89] | 1.27*  [1.01, 1.60] | 1.18  [0.96, 1.44] | 1.02  [0.88, 1.19] | 1.09  [0.88, 1.35] |
| Marital status |  |  |  |  |  |
| Currently married [ref] | - | - | - | - | - |
| Previously married | 0.74*  [0.59, 0.92] | 0.84  [0.69, 1.03] | 0.91  [0.74, 1.12] | 0.89  [0.75, 1.06] | 0.76*  [0.84, 0.92] |
| Living with a partner | 0.65*  [0.48, 0.88] | 0.83  [0.60, 1.14] | 0.95  [0.71, 1.27] | 0.76*  [0.59, 0.97] | 0.86  [0.64, 1.11] |
| Never married | 0.82*  [0.68, 0.99] | 0.88  [0.72, 1.08] | 1.00  [0.85, 1.33] | 0.81*  [0.67, 0.99] | 0.81*  [0.67, 0.96] |
| Bodyweight^2^ |  |  |  |  |  |
| Healthy [ref] | - | - | - | - | - |
| Overweight | 1.05  [0.85, 1.31] | 0.90  [0.72, 1.12] | 1.01  [0.84, 1.22] | 0.90  [0.75, 1.07] | 1.06  [0.84, 1.33] |
| Obese | 0.90  [0.65, 1.25] | 0.86  [0.72, 1.02] | 0.96  [0.79, 1.16] | 0.77*  [0.62, 0.95] | 1.01  [0.81, 1.28] |
| Poverty income ratio |  |  |  |  |  |
| < 130% FPL | 0.80*  [0.66, 0.96] | 1.31*  [1.11, 1.54] | 0.96  [0.79, 1.16] | 1.17  [1.00, 1.36] | 1.14  [0.97, 1.33] |
| ≥ 130% FPL [ref] | - | - | - | - | - |

Note: Multivariate regression was used to adjust for sex, race/ethnicity, age, education, marital status, poverty, body-weight category and engagement in weight loss activities.

*Odds Ratio significant at p<0.05.
